# Editorial: Identifying the Interdisciplinary Determinants, Biologic Mechanisms, and Best Practices for the Prevention and Elimination of Minority Health Disparities

**DOI:** 10.3389/fpubh.2022.863011

**Published:** 2022-03-18

**Authors:** Allison A. Appleton

**Affiliations:** Department of Epidemiology and Biostatistics, University at Albany School of Public Health, Rensselaer, NY, United States

**Keywords:** health disparities, interdisciplinary, multilevel determinants, biological mechanism, social determinants, environmental determinants, research and practice

From macro-level social processes, to environmental exposures, and molecular-level alterations, the causes of minority health disparities are varied and complex. To that end, the National Institute of Minority Health Disparities has developed a multilevel research framework that considers biologic, behavioral, environmental (physical, social, cultural), and health care system factors as jointly shaping health outcomes and health disparities (1). While comprehensive in its scope, minority health disparities research and practice often focus on singular components of the framework in part due to our discipline specific training. As the causes of minority health disparities cross disciplines, so too must investigator expertise and practice-based solutions. Moreover, in order to eliminate health disparities, research and practice should move beyond focus on proximal health care related determinants, and more broadly consider upstream contextual, social, and environmental causes. Also, the biologic mechanisms connecting the exogenous determinants to health disparities are not well-understood. Biologic factors that are sensitive to lived experience may be key in understanding how deleterious exposures become biologically embedded to drive health disparities. Thus, a critical interdisciplinary review of the many factors that contribute to minority health disparities is warranted. Doing so will help to identify gaps in the evidence base and highlight next steps for research and practice.

In this Research Topic, we present a collection of articles that focus on the causes of health disparities from both social and environmental perspectives, as well as innovative solutions. For example, in their review of energy insecurity (defined as the inability to meet household energy needs, such as heat and electricity), Jessel et al. discuss the adverse health consequences of unaffordable and inadequate household energy in the context of climate change for vulnerable populations. They note that energy insecurity co-occurs with, and is exacerbated by, other social determinants of health like socioeconomic status, race, and place. Such intersectional inequality is also discussed in Taylor et al. review of the multilevel determinants of HIV testing disparities among foreign born black men living in the United States. The authors discussed not only how individual, community and policy level factors can each thwart access to HIV testing, but also the accumulated impact of these factors along with having multiple stigmatized identities (e.g., race, gender, sexual orientation, addiction) work to exacerbate risk among this population. In terms of solutions and strategies to prevent and eliminate health disparities, Gómez et al. describe the *Juntos por la Salud* (JPLS; Together for Health) initiative, which is a mobile health and wellness program for Mexican immigrant population living in 11 metropolitan areas in the United States. Mexican migrants experience a myriad of significant social and socioeconomic risks, including low educational attainment and low pay occupations, as well as high psychosocial stress related to immigration status (e.g., fear of deportation) and discrimination, which in turn results in low health care utilization and poor health outcomes. JPLS works to reduce barriers to health care for this population by providing culturally sensitive and linguistically matched health education and services for free. Taken together, these studies indicate that as the causes of health disparities are multifaceted and multilevel, so too must be the interventions.

The Research Topic also includes empirical papers that focused explicitly on biologic mechanisms—epigenetic alterations and telomere length—linking adversity to health outcomes. Epigenetic alterations, often measured by DNA methylation, can modulate gene expression and influence health outcomes. Epigenetic alterations are sensitive to adverse exposures commonly experienced by vulnerable populations, and can be altered during sensitive developmental periods like pregnancy, thereby possibly contributing to the biologic embedding and intergenerational transmission of health disparities (2, 3). In DeLano et al. study of pregnant women and their infants, higher levels of community socioeconomic deprivation during pregnancy was associated with significant epigenetic change to a gene involved in the infant's stress response system (*SLC6A4*), suggesting possible future developmental risk for the child. Similarly, telomere length (which refers to the structures at the end of chromosomes which shorten with age) is considered a biomarker of aging and research has shown telomere shortening and concomitant disease risk can be accelerated through experiences of stress and social adversity (4). In a nationally representative US sample of Hispanic adults, Ishino et al. found variation in telomere length according to different profiles of acculturation (e.g., low acculturation, assimilated, integrated), suggesting stress related to acculturation over the life course may impact biology.

Finally, our Research Topic includes timely papers related to disparities and the COVID-19 pandemic. For example, Strully et al. take up the critical point of COVID-19 vaccine hesitancy among racial and ethnic minorities, who have been disproportionately impacted by the pandemic. Using data collected from minority populations in New York State, Strully et al. discuss the legacy of historical health injustice and ongoing inequities that fuel continued medical distrust, as well as the need for vaccine campaigns to rely on trusted community voices and community assets to promote vaccination. The associated commentary paper by Teo, as well as the perspective paper by Ullah et al. discuss a COVID-19 future, both in terms of data needs for promoting vaccination uptake, and impacts of the pandemic on future birth rates due to changes in economic conditions, mental health, and mortality.

Taken together, the papers in this Research Topic represent the interdisciplinary and innovative thinking that is necessary to effectively address and eliminate health disparities. Moreover, in addition to the myriad of risks that jointly contribute to disparities, we must also consider community and individual level assets that can promote health and resilience. Wu et al. paper on sleep duration and ideal cardiovascular health (5) is one example of this positive approach. It is our hope that this collection of papers will lay stimulate collaborations across disciplines and lay the foundation for future minority health disparities research and practice.

## Author Contributions

AA conceptualized, drafted, edited, and finalized this editorial.

## Conflict of Interest

The author declares that the research was conducted in the absence of any commercial or financial relationships that could be construed as a potential conflict of interest.

## Publisher's Note

All claims expressed in this article are solely those of the authors and do not necessarily represent those of their affiliated organizations, or those of the publisher, the editors and the reviewers. Any product that may be evaluated in this article, or claim that may be made by its manufacturer, is not guaranteed or endorsed by the publisher.
